# 

**Published:** 1890-02-01

**Authors:** 


### NEW OFFICES IN THE STRAND.

*On and after February 1st,* 1890, The Hospital *will be published at* **140, Strand, W.C.** *It has been found desirable to establish* The Hospital *in this central and convenient locality, owing to the rapid extension of the business of this paper of late.*

### NOTICE TO CORRESPONDENTS.

All MS., Letters, Books for Review, and other matters intended for the Editor, should be addressed The Editor, The Lodge, Porchester Square, London, W.

### Notice to Advertisers and Subscribers.

All Advertisements, Orders for Copies of The Hospital, and Business Communications should be addressed to The Publisher, *not to the Editor,* The Hospital, 139-140, Salisbury-court, Fleet-street, E.C.

### Bound Volumes of "The Hospital."

Vol. VI., containing full page portraits of T.R.H. the Prince and Princess of Wales, and Miss Florence Nightingale, is now ready, 4s., post free. Cases for binding, may be had of the Publisher, at 1s. 6d. each.

### To Residents, Secretaries, and Matrons

The Editor will be glad to have the Regulations and each Report as issued by Asylums, Hospitals, Convalescent and Nursing Institutions, as well as those of other Charities, and an early copy in duplicate of all Appeals and Festival Notices, etc., addressed to *The Editor, The Lodge, Porchester-square, London, W.* Any superintendent, secretary, matron, or other chief official who regularly sends such information may be registered, on application to the Publisher, to receive free of cost a cover for binding The Hospital in half-yearly volumes.

### Scraps and Gleanings.

A horse died of hydrophobia at Otley last week.

It is proposed to appoint Dr. Bennett aural surgeon to Leicester Infirmary.

The election of matron at St. Thomas's Hospital will take place on Feb. 12th.

About 12,000,000 children in the United States are taught by law the nature and effects of intoxicants.

Mr. Cheetham has been elected hon. sec. of Rochdale Infirmary, in place of Mr. Molesworth, resigned.

Liverpool Northern Hospital is somewhat deeply in debt, and further subscribers are badly needed.

An excellent conversazione in aid of the Ballymena Cottage Hospital was lately held in the Protestant Hall.

The Great Northern Central Hospital has received the additional present of ten pheasants from the Queen.

Tiverton Infirmary congratulates itself on having an economical matron, for its list of subscribers is very low.

Belfast Samaritan Hospital for Women treated 96 in-patients last year. There were four cases of ovariotomy.

Glasgow Eye Infirmary treated over 15,000 patients last year. Students to the number of 47 attend the classes.

The Clothworkers' Company have given a donation of £2,000 towards the establishment of a technical institute in North London.

Yarmouth Cottage Hospital treated 21 in and 96 out patients during the year. A new building was opened last August.

Sir Edwin Chadwick, K.C.B., has subscribed £5 to the Church of England Burial, Funeral, and Mourning Reform Association.

The Queen has sent a chest of cast linen and 20 pheasants as a present to the Seamen's Hospital (late "Dreadnought") Greenwich, S.E.

Surgeon Morse, Surgeon-Major Rogers, and Surgeon-Major Hayes have been decorated with the 3rd class of the Medjidieh by the Khedive.

In the Birmingham city hospital the number of cases of scarlet fever is 369, as compared with 484, the maximum number during the epidemic.

Over £2,000 has been collected to build a children's ward for Burnley Victoria Hospital, chiefly through the energy of Mrs. and Mr. Edward Ecroyd.

The Secretary of the Stanley Hospital, Liverpool, has received £1,000 from the Roger Lyon Jones Fund for the endowment fund of the hospital

Dr. Jayne, Bishop of Chester, distributed certificates to successful competitors of the ambulance class of the Chester Hospital Saturday Committee.

Frome Cottage Hospital reports continued progress, the number of patients treated having risen from 111 in 1888 to 130 in 1889. There is a balance in hand.

Finsbury Dispensary last year received subscriptions from the King of the Belgians and the Marquis of Northampton. Over 18,000 new cases were treated.

The site for the new Halifax Infirmary has been secured at a cost of £13,798. Contributions promised to the building fund amount to £54,000. Bravo, Halifax!

Mrs. E. B. Carr-Gomm (Lady of the Manor of Rotherhithe) laid the foundation-stone of the new buildings for the South Bermondsey Club and Institute, on Saturday.

Turner Memorial Hospital, at Keith, treated 42 patients last year. The income was £258, and the expenditure £343, of which over £100 went in building expenses.

Some years ago the Emperor of Austria brought on a bad gastric derangement by over-indulgence in Virginias, and the doctors have since cut down his allowance to five a day.

Captain Leggett presided at the annual meeting of Worcester Ophthalmic Hospital. The report was in every way satisfactory, 61 in and 685 out-patients having been treated.

The Doll Show which Mrs. Garrett Anderson and others are getting up, in aid of the new Hospital for Women, will have classes for dolls dressed as nurses and dolls dressed as patients.

A man named Loughan was found dead in his bed in a lodging-house in Dungannon. He got a bottle of cough mixture from a local chemist, to be taken in 23 doses, but he drank the whole of it at once.

The rats in the Fen districts of Lincolnshire have attacked the potato and mangold graves, and the young plants in the field. A French chemist has offered to come over and exterminate the vermin.

A Cornish giant, named Mr. Jacob Corin, innkeeper, of Penzance, who weighed 334 pounds, and who died of congestion of the lungs, was buried recently. The coffin was 7ft. 4in. in length, 3ft. 1¾in. in breadth, and 2ft. 3½in. in depth.

The St. John Ambulance Association has opened an ambulance station under the west portico of St. Paul's Cathedral, where a qualified attendant will be on duty every day, except Sunday, from nine a.m. to six p.m., provided with first-aid appliances and means of transport.

At the 101st annual meeting of the City Dispensary, Mr. F. P. Alliston, C.C., who presided, said there were 1,015 on the books on December 31st. He said they wanted money, as they had very little invested funds. Out of the £1,500 which they spent they had to raise £1,400 every year.

A scheme has been formulated by a committee of experts, acting on behalf of the London County Council, for establishing a new hospital in the metropolis for the treatment of the insane, and for the scientific study of insanity. It is estimated that the cost of erecting a hospital with 100 beds would be £32,000.

